# Monitoring and Assessment of Airborne Respirable Limestone Dust and Free Silica Content in an Indian Mine

**DOI:** 10.5696/2156-9614-9.23.190904

**Published:** 2019-08-06

**Authors:** Priyanka Mankar, Bibhuti B. Mandal, Debasis Chatterjee

**Affiliations:** 1 National Institute of Miners' Health, Nagpur, India; 2 Department of Mining Engineering, Indian Institute of Technology Kharagpur, Kharagpur, India

**Keywords:** respirable dust, free silica, time-weighted average (TWA), Fourier transform infrared (FTIR), open cast mine, Director General of Mines Safety (DGMS)

## Abstract

**Background.:**

Dust exposure and its related harmful effects on miners is a serious health issue.

**Objectives.:**

The present study was undertaken to identify respirable dust concentrations and free silica content in 24 dust samples collected from a limestone mine during pre-monsoon and post-monsoon seasons.

**Methods.:**

Time-weighted average (TWA) dust concentrations were calculated for 8-hour work shifts followed by determination of free silica concentration using the Fourier transform infrared spectroscopy technique.

**Results.:**

The TWA dust concentration for personal and area dust samples during September 2013 was found to be in the range of 0.32–1.04 mg/m^3^ and 0.25–0.54 mg/m^3^, respectively. For February 2014, the TWA dust concentration was between 0.62–1.23 mg/m^3^ for personal samples and 1.04–2.64 mg/m^3^ for area samples. Samples collected in February 2014 had marginally higher values of dust levels in the air of the mine compared to September 2013. The highest dust concentration was found to be 1.23 mg/m^3^ for a drill operator and 2.64 mg/m^3^ at the crusher belt conveyor junction. The average free silica percent for the samples collected in February 2014 was 1.73%, which was almost double compared to September 2013 (0.87%).

**Conclusions.:**

In both seasons, personal dust samples had higher free silica content than area dust samples. Even though dust concentrations were below the permissible limit, workers had widely different exposures, hence many of them may be at risk of pneumoconiosis when exposure is prolonged.

**Participant Consent.:**

Obtained

**Competing Interests.:**

The authors declare no competing financial interests.

## Introduction

India is ranked second in terms of total worldwide limestone production after China, with 313.2 million tons produced from 2016–17.^1^ Limestone is considered the world's most versatile mineral.^1^ India has large limestone resources distributed over different parts of the country. It is an important raw material for various industries, such as the construction industry, steel production, cement manufacturing, etc. Cement and steel have played a crucial role in infrastructure growth in India. From 2016–17, cement grade limestone accounted for 97% of the total limestone production, followed by 2% iron and steel grade and 1% chemical grade.^1^ Limestone is a calcareous sedimentary rock mainly composed of carbonates. Calcite and dolomite are the major mineral components of limestone. Limestone frequently contains magnesium carbonate, either as dolomite or magnesite mixed with calcite, becoming ‘dolomitic’ or ‘magnesian’ limestone. Limestone containing a minimum of 45% calcium oxide is generally used in cement manufacture.

Most major mining activities directly or indirectly contribute to air pollution.^2^ Operations such as drilling, blasting, crushing, milling, screening, conveying, transporting, etc. are primarily responsible for dust generation in mines. Previous studies have explored the impacts on the immediate environment and health of workers due to mining and processing of limestone.^3^ Doig reported eight cases of pneumoconiosis in limestone workers who were involved with crushing and grinding for 16 to 39 years.^4^ X-ray and other examination of workers showed symptoms of dyspnoea, cough, sputum, chest pain, asthmatic attack, weight loss, etc.^4^ He also concluded that workers involved with the manufacturing and construction industry and mining were more highly exposed to crystalline silica.^4^ Koelsch and Kaestle examined 82 workers in a shell limestone mine and concluded that limestone can cause pulmonary changes over a prolonged period under certain conditions.^4^ Gardner reported that calcium carbonate dust can help decrease the toxicity of quartz.^4^ Landwehr *et al.* stated that rocks with greater calcium mineral content are less dangerous and the associated dust is known as ‘protestor dust.’^4^

### Silica exposure

Generation of respirable silica dust during mining is a major concern due its abundance in most mineral work. Millions of workers around the world are exposed to silica dust. Limestone rock contains crystalline silica in different quantities depending upon the type of rock.^5^ Limestone contains up to 40% of crystalline silica in some cases. Silica dust exposure occurs more in certain occupations, such as mining, sandblasting, stone cutting, surface drilling, silica flour mill operations, etc. Exposure to silica may decrease resistance to infection by facilitating viral and bacterial contamination through the respiratory tract.^6^ Disease severity depends on the amount of crystalline silica in the respirable dust that is deposited in the lungs.^7^

Previous studies provide strong evidence for health problems related to long term airborne silica exposure. For example, Cauda *et al*. revealed that occupational risk from crystalline silica develops from long term exposure to respirable silica dust which can lead to a potentially fatal lung disease (silicosis).^8^ Hnizdo and Vallyathan found that severe silicosis can also cause significant lung impairment. They reported that the risk of developing tuberculosis was greater in workers exposed to silica dust even in the absence of silicosis.^9^ Healy *et al*. studied respirable crystalline silica exposure in a group of stone workers, i.e. stone cutters and stonemasons who work on sandstone, limestone, lime mortar and granite involved in the restoration and maintenance of heritage buildings in Ireland. The results showed that stone workers grinding and cutting sandstone had very high levels of respirable crystalline silica exposure.^10^ In cohort studies, Chen *et al*. found a significant exposure response relationship between silica dust and increased mortality among Chinese workers due to cardiovascular disease, even at low dust concentrations.^11^ Yassin *et al*. also confirmed an association between other health problems due to airborne silica exposure, including chronic bronchitis, chronic obstructive pulmonary disease, lung cancer, airflow obstruction, rheumatoid arthritis, scleroderma, Sjogern's syndrome, lupus, and renal disease.^12^

**Abbreviations**
*DGMS*: Director General of Mines Safety
*FTIR*: Fourier transform infrared
*TWA*: Time weighted average

The present study was undertaken to identify respirable dust concentrations and free silica content in 24 dust samples collected from a limestone mine during pre-monsoon and post-monsoon seasons.

## Methods

The present study estimated the exposure of workers by personal dust samplers, evaluated concentrations of respirable dust at the workplace by area monitoring and determined the free silica content in dust samples collected during the study. Lastly, the results were compared with the guidelines from the Metalliferous Mines Regulations, 1961 to draw inferences.

### Study area

The airborne respirable dust survey was carried out in the east Indian state of Jharkhand. The study area is a well-known open cast limestone mine which manufactures raw material for cement production. It is the only company in India to exploit low-grade limestone for cement manufacture after beneficiation. It is one of largest producing cement mines in India with a production capacity of about 1.8 million tons per annum and a total lease area of 662.75 hectares. The area lies between 220°44′35″N to 220°25′10″N latitude and 850°43′50″E to 850°44′35″E longitude. This region is mainly comprised of rocks of the Kolhan Group, Singhbhum granite, and rocks of the Iron Ore Group. The area where the production and limestone mine is located forms a part of the Chotanagpur Plateau. The limestone belongs to the Kolhan series of the Dharwar age. As mining operations reach the limestone overburden, the underling Kolhan sandstone is degraded. The limestone varies in color, including off-white, pink, and dark brown.

Mining work is carried out over 24 hours a day in three shifts. Shift A operates between 06:00 am to 02:00 pm, shift B operates between 02:00 pm to 10:00 pm and shift C operates between 10:00 pm to 06:00 pm. There is an additional (overlapping) shift for various administrative and general maintenance work, which operates between 07:30 am to 05:00 pm. The types of mining machinery available at the mine are shown in Table 1.

**Table 1:** Machinery Available in Mine

**Serial number**	**Type of machinery**	**Capacity**	**Number of units**
1	Shovel	3.5 m^3^	2
		4.1 m^3^	5
2	Drilling machine	152 mm	4
		115 mm	1
3	Dumper	35 tons	13
		40 tons	2
4	Dozer	—	3
5	Backhoe loader	3.5 m^3^	2

### Sampling

Respirable dust sampling (area and personal sampling)was carried out using a Director General of Mines Safety (DGMS) approved dust sampler, Sidekick 51Ex (SKC, UK), following DGMS sampling guidelines, from February 2014 (pre-monsoon period) to September 2013 (post-monsoon period) at specific locations in the mine. Twelve dust samples were initially collected in September 2013 (5 area samples (AD) and 7 personal samples (PD)), followed by another field study in February 2014 (6 area samples (AD) and 6 personal samples (PD)) at different locations in the mine after consultation with mining officials. The dust samples for both seasons were collected in A and B shifts for 6–7 hours (355–465 minutes each) during active production phases in the mine. Three area and four personal dust samples in A shift and two area and three personal dust samples in B shift were collected in September 2013. In February 2014, three area and three personal dust samples in A shift and three area and three personal dust samples in B shift were collected. Analysis was performed on all personal and area dust samples collected during both study periods. Sidekick51Ex has a correlation factor of 1.13 with the Mine Research Establishment 113A gravimetric dust sampler (reference sampler). Hence the results obtained by this sampler were divided by 1.13 to get the equivalent Mine Research Establishment concentration in mg/m.^3^

The sampling assembly consisted of a 37mm cassette filter holding polyvinyl chloride filter paper of 5.0-μm pore, aluminum cyclone and coupler. Each polyvinyl chloride filter paper was pre- and post-weighed on a digital balance (Shimadzu, AW-220). Pre- and postcalibration of Sidekick51Ex samplers was carried out with a DryCal DC-Lite (Bios International, USA) calibrator for a flow rate of 2.2 liters/min. Source monitoring (area sampling) was carried out continuously during the entire working shift during active operations. For *area sampling* the Sidekick51Ex sampler was kept at a distance of 5 to 15 m away from the workers face and positioned vertically above the ground at breathing level, directed down wind. For *personal sampling*, the sampler was attached to the worker from the time he entered the mine to the time he left the mine, otherwise known as portal to portal sampling. The Sidekick51Ex was attached to the worker's belt and the cyclone sampler along with cassette was attached to the collar (near the breathing zone) of the worker.

The respirable dust collected on polyvinyl chloride filter paper was also used for estimation of free silica by Fourier transform infrared (FTIR) spectroscopy (Bruker, USA). Sample preparation was done by digesting the collected dust samples in the muffle furnace for four hours at 600°C followed by mixing it thoroughly with potassium bromide (0.2g) in a mortar and pestle. The mixture was transferred to a 13 mm pellet die and the uniform pellets were prepared using a hydraulic pellet press. The percentage of free silica was then quantitatively assessed using OPUS software on the FTIR.

### Study subjects

The present study was conducted on a total of twenty four male workers (only males were working in the mine, and only 24 miners were available—all consented) engaged with the different heavy earth moving machines (HEMMs) in the mine during both the periods. All workers were aged between 30 and 45. Worker participation was voluntary, and informed consent was obtained.

The study was conducted on the workers after consultation and coordination with the mining officials. This type of air quality sampling is considered part of a miner's job under occupational hygiene activities. The workers were exposed for 6–7 hours during the active production phase of the mine in Sept. 2013 and Feb. 2014.

## Results

The time weighted average (TWA) for dust concentration was calculated in 8-hour shifts and the concentration values (mg/m^3^) for personal dust samples are given in Table 2.

**Table 2:** Dust Concentration of Personal Dust Samples (n=7) Collected in September 2013

**Serial number**	**Sample ID**	**Location**	**Operation**	**8-hour TWA dust concentrations (mg/m^3^)**
1.	PD1	BC1 to BC2 belt clearing operator	Transfer ore from BC1 to BC2	0.43
2.	PD2	BC2 to pipe conveyer operator	Transfer ore from BC2 to pipe conveyor	1.04
3.	PD3	At RL280 bottom pit	Shovel Tata Hitachi EX500 operator	0.37
4.	PD4	Bottom pit RL 280	Dumper BEML 210M operator	0.43
5.	PD5	Pit floor RL 280	Shovel Tata Hitachi EX 600,number 7 operator	0.39
6.	PD6	Bench Number 1 RL292	Drill Copco Flexi ROC D60, helper	0.32
7.	PD7	RL280 to crusher hopper	Dumper Komatsu HD465 operator, transfer iron ore from RL280 to crusher hopper	0.40

Abbreviations: PD, personal dust; BC, belt conveyor; RL, reduced level;

Table 2 shows the dust exposure of workers at different locations in the mine. The dust exposures of individuals working near belt conveyors were affected due to movement of dumpers in the vicinity. It was observed that 8-hour TWA concentrations of personal respirable dust at all sampled locations were well within the permissible limit (i.e. below 3 mg/m^3^) as recommended by DGMS. The area of belt conveyor 2 to pipe conveyer operator had the highest dust exposure of 1.04 mg/m^3^. The TWA dust levels ranged from 0.32 to 1.04 mg/m.^3^

Table 3 shows the 8-hour TWA dust concentration (mg/m^3^) for area dust samples collected during different mining operations at their respective locations. It was observed that 8-hour TWA dust concentration levels at all the sampled locations were well below the permissible limit (i.e. below 3 mg/m^3^). The crusher hopper area showed the highest dust level of 0.54 mg/m^3^ compared to others. Dust concentrations at other locations ranged between 0.25 to 0.54 mg/m^3^.

**Table 3:** Dust Concentration of Area Dust Samples (n=5) Collected in September 2013

**Serial number**	**Sample ID**	**Area/location**	**Operation**	**8-hour TWA dust concentration (mg/m^3^)**
1.	AD1	Crusher hopper area	Dumper unloads ore onto crusher hopper	0.54
2.	AD2	Quarry crusher	Crushing of limestone	0.25
3.	AD3	BC2 to pipe conveyer junction	Transfer of limestone from belt conveyor to pipe conveyor	0.27
4.	AD4	Haul road near view point approaching crusher hopper	Movement of dumpers and other service vehicles	0.25
5.	AD5	Mine bottom pit haul road junction	Movement of dumpers and other service vehicles	0.33

Abbreviations: AD, area dust; BC, belt conveyor.

Tables 4 and 5 gives the silica content (%) of all respirable dust samples collected in September 2013. Doublet peak characteristic of free silica in FTIR was observed during analysis of all personal and area dust samples. However, the percentages of free silica content in all the dust samples were below 5%. The highest percentage of free silica (1.68%) was observed in ‘Personal Dust 6’ which was the helper operating near the drill machine. This was closely followed by the samples ‘Personal Dust 7’ (1.42%) and ‘Personal Dust 4’ (1.15%) where the operators were engaged in driving dumpers on the haul roads to transport ore from the pit bottom to crusher hopper *(Table 4).* Likewise, for area dust samples, the maximum was observed in location Area Dust 2 at the quarry crusher with 1.18% of free silica followed by the sample Area Dust 1 at the crusher hopper area (1.06%) *(Table 5).*

**Table 4:** Free Silica Content (%) in Personal Dust Samples Collected in September 2013

**Serial number**	**Sample ID**	**Weight of free silica (mg)**	**Weight of dust (mg) (W2-W1)**	**Free silica content (%)**
1.	PD1	0.0035	0.54	0.65
2.	PD2	0.0059	1.24	0.47
3.	PD3	0.0036	0.44	0.82
4.	PD4	0.0059	0.51	1.15
5.	PD5	0.0042	0.47	0.88
6.	PD6	0.0064	0.38	1.68
7.	PD7	0.0068	0.48	1.42

Abbreviations: W1, initial weight; W2, final weight; PD, personal dust.

**Table 5:** Free Silica Content (%) in Area Dust Samples Collected in September 2013

**Serial number**	**Sample ID**	**Weight of free silica (mg)**	**Weight of dust (mg)(W2-Wl)**	**Free silica content (%)**
1.	AD1	0.0068	0.64	1.06
2.	AD2	0.0034	0.29	1.18
3.	AD3	0.0019	0.32	0.59
4.	AD4	0.0020	0.30	0.67
5.	AD5	0.0019	0.39	0.49

Abbreviations: W1, initial weight; W2, final weight; AD, area dust.

The degree of respirable dust exposure of workers at various locations is shown in Table 6. The 8-hour TWA concentration of all personal respirable dust samples were within the permissible limit (i.e. below 3 mg/m^3^) as stipulated by the DGMS. The drill operator (Personal Dust 13) at the safety middle bench had the highest dust concentration (1.23 mg/m^3^), which was close to 50% of the permissible limit (just below 1.5 mg/m^3^). This was followed by the backhoe operator (Personal Dust 9) working at purple shell top bench (west side) with a dust exposure level of 1.04 mg/m^3^. The rest of the samples showed dust concentrations below one percent (1%).

**Table 6:** Dust Concentration of Personal Dust Samples (n=6) Collected in February 2014

**Serial Number**	**Sample ID**	**Location**	**Machine and activity**	**8-hour TWA dust concentration (mg/m^3^)**
1.	PD8	Purple shell, top bench (east side)	L&T Kumatsu, back hoe operator number 1, loading ROM onto tippers	0.80
2.	PD9	Purple shell, top bench(west side)	L&T Kumatsu, back hoe, number 2 operator, loading overburden onto tippers	1.04
3.	PD10	Purple green shell, top bench (east side)	Tata Hitachi L&T, number 7 operator	0.85
4.	PD11	Purple green shell, top bench (east side)	Dumper Komatsu HD465 operator	0.66
5.	PD12	Purple shell, (south side)	Dumper Komatsu HD465 operator	0.62
6.	PD13	Safety middle bench	Drill IR -5, CM 260 operator, drilling operation	1.23

Abbreviations: PD, personal dust ROM, run-of-mine.

The 8-hour TWA dust concentrations (mg/m^3^) for different locations sampled in the mine are presented in Table 7. Although the 8-hour TWA concentrations at all the sampled locations were within the acceptable limit, the transfer point from crusher belt conveyor 1 to belt conveyor 2 generated a dust level of 2.64 mg/m^3^ which exceeded 75% of the acceptable limit (i.e. 2.25 mg/m^3^). Locations ‘Area Dust 11’ (belt conveyor 2 to pipe conveyor junction), and ‘Area Dust 7’ (drilling area at the purple shell top bench) had dust concentrations of 1.52 mg/m^3^, both of which were above 50% of the acceptable limit (i.e. 1.5 mg/m^3^). In addition, the loading operation at purple shell top bench in the mine had a dust concentration of 1.42 mg/m^3^, which was nearly 50% of the acceptable limit. The dust levels observed for the locations at purple shell haul road and the crushing hopper area were 1.14 mg/m^3^ and 1.04 mg/m^3^, respectively.

**Table 7:** Dust Concentration of Area Dust Samples (n=6) Collected in February 2014

**Serial Number**	**Sample ID**	**Area/location**	**Operation**	**8-hour TWA dust concentration (mg/m^3^)**
1.	AD6	Purple shell haul road	Movement of dumpers, tippers and mine vehicles	1.14
2.	AD7	Drilling area, purple shell top bench	Drilling operation	1.52
3.	AD8	Purple shell top bench	Shovel loading overburden onto tipper	1.42
4.	AD9	Crushing hopper area	Dumper loading ore onto tipper	1.04
5.	AD10	Crusher belt conveyor junction BC1 to BC2	Transfer of ore from one conveyor to another	2.64
6.	AD11	BC2 to pipe conveyor junction	Transfer of ore from belt conveyor to pipe conveyor	1.52

Abbreviations: AD, area dust BC, belt conveyor.

Tables 8 and 9 show the results of the free silica content (%) of personal and area dust samples collected in the mine during February 2014. A total of six personal and six area dust samples were qualitatively and quantitatively analyzed for free silica (quartz) at 798 cm^−1^ wave number by FTIR spectroscopy. Although the typical doublet peak characteristic of free silica was observed for all the personal and area respirable dust samples, the percentage of free silica content was below 5%. Among the workers engaged in various operations, the dust sample of drill operator, ‘Personal Dust 13’, was found to contain the highest percent of free silica i.e. 2.08%. Similarly, samples of dumper operators of Kumatsu (‘Personal Dust 11’) and Kumatsu K2 (‘Personal Dust 12’) had a free silica content of 1.60% and 1.31%, respectively. Across the locations, the highest free silica content was observed in the sample collected at the drilling site (‘Area Dust 7’). The samples from the crusher hopper site (‘Area Dust 9’) and crusher belt conveyor junction (‘Area Dust 10’) had lower silica content, at 1.67% and 0.99%, respectively.

**Table 8:** Free Silica Content (%) in Personal Dust Samples Collected in February 2014

**Serial Number**	**Sample ID**	**Weight of free silica (mg)**	**Weight of dust (mg) (W_2_-W_1_)**	**Free silica content (%)**
1.	PD8	0.0057	0.95	0.67
2.	PD9	0.0095	1.10	0.86
3.	PD10	0.0065	0.90	0.73
4.	PD11	0.1120	0.70	1.60
5.	PD12	0.0085	0.65	1.31
6.	PD13	0.0270	1.30	2.08

Abbreviations: W1, initial weight; W2, final weight; PD, personal dust.

**Table 9:** Free Silica Content (%) in Area Dust Samples Collected in February 2014

**Serial Number**	**Sample ID**	**Weight of free silica (mg)**	**Weight of dust (mg) (W_2_-W_1_)**	**Free silica content (%)**
1.	AD6	0.0059	1.20	0.49
2.	AD7	0.0323	1.60	2.02
3.	AD8	0.0059	1.50	0.39
4.	AD9	0.0184	1.10	1.67
5.	AD10	0.0277	2.79	0.99
6.	AD11	0.0064	1.60	0.40

Abbreviations: W1, initial weight; W2, final weight; AD, area dust.

Table 10 presents a comparison of how the silica content/percent varied for area and personal samples and both categories in terms of mean, range, weight of free silica content, sum of actual weight of dust and percent of silica content observed through the pre-monsoon and post-monsoon periods. For September 2013, the total quantity of dust in personal samples (4.06 mg) and personal mean silica content in the dust samples (0.0052 mg) were higher compared to those of area dust samples (1.94 mg and 0.0032 mg, respectively). In addition, the free silica percentage was higher in personal samples compared to area samples. The percentage of free silica content for combined (personal and area) samples was 0.87%, which can be considered to be a representative figure for the entire mine.

**Table 10:** Free Silica Content (mg/m^3^) in Personal and Area Samples During September 2013 and February 2014

**Duration**	**Samples**	**Number of samples**	**Mean and range of free silica content (mg)**	**Sum of weight of free silica content (mg)**	**Sum of weight of dust (mg) (W_2_-W_1_)**	**Free silica content(%)**
**September 2013**	**Personal**	07	0.0052 (0.0035–0.0068)	0.0363	4.06	0.89
	**Area**	05	0.0032 (0.0019–0.0068)	0.0160	1.94	0.83
	**Personal + Area**	12	0.0044 (0.0019–0.0068)	0.0523	6.00	0.87
**February 2014**	**Personal**	06	0.0282 (0.0057–0.1120)	0.1690	5.60	3.02
	**Area**	06	0.0161 (0.0059–0.0323)	0.0966	9.79	0.99
	**Personal + Area**	12	0.0222 (0.0057–0.1120)	0.2658	15.39	1.73

For the samples collected during February 2014, the free silica percent analyzed in personal samples was much greater than area samples. The percent of free silica content for all samples combined was found to be 1.73%.

Table 11 summarizes and evaluates dust concentrations in terms of minimum, maximum, average, and concentrations at ≥50% and ≥75% of permissible limits from the results of monitoring in September 2013 (7 personal and 5 area dust samples) and February 2014 (6 personal and 6 area respirable dust samples). Operators at various locations in the mine were working with a personal dust exposure range between 0.32–1.04 mg/m^3^ with an average concentration of 0.48 mg/m^3^ in September 2013, and 0.62–1.23 mg/m^3^ with an average of 0.87 mg/m^3^ in February 2014. Mine workers were exposed to higher dust concentrations in February 2014 compared to September 2013. The dust survey through February 2014 revealed that one worker and three area locations were found to have dust concentrations above 50% of the permissible limit and dust concentrations exceeded 75% of the limit prescribed by the DGMS in one location.

**Table 11:** Respirable Dust Concentration (mg/m^3^) in Personal and Area Samples During September 2013 and February 2014

**Duration**	**Samples**	**Number**	**Minimum (mg/m^3^)**	**Maximum (mg/m^3^)**	**Average (mg/m^3^)**	**Number and concentration of dust samples ≥ 50%**	**Number and concentration of dust samples ≥ 75%**
**September 2013**	**Personal**	7	0.32	1.04	0.48	0	0
	**Area**	5	0.25	0.54	0.33	0	0
**February 2014**	**Personal**	6	0.62	1.23	0.87	n=l 1.23 mg/m^3^	0
	**Area**	6	1.04	2.64	1.55	n=3 1.42mg/m^3^, 1.52mg/m^3^ 1.52mg/m^3^	n=l 2.64 mg/m^3^

Figure 2 and 3 illustrate the exposure of miners with respirable dust concentrations in various sites in the mine in September 2013 and February 2014, respectively.

**Figure 1:**
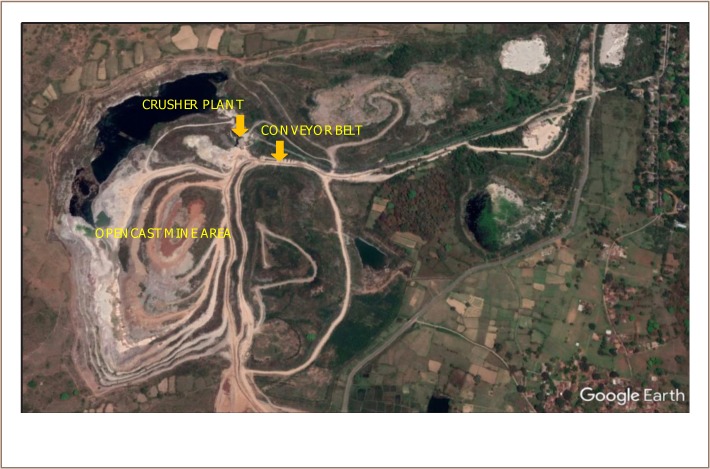
The Mine Study Site

**Figure 2:**
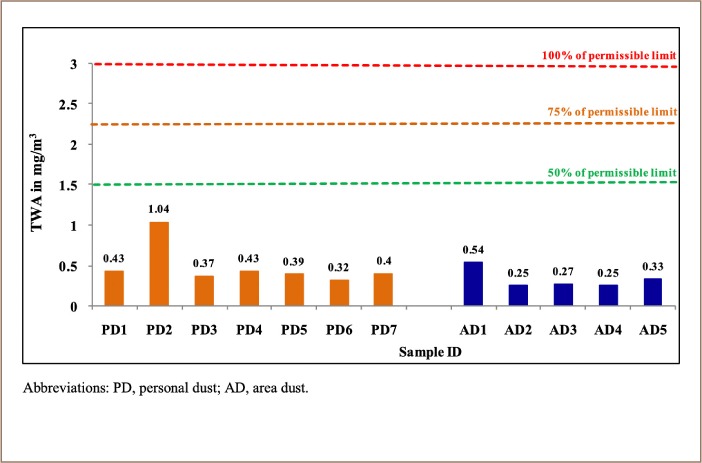
Personal and Area Dust Exposure Monitoring During September 2013

**Figure 3:**
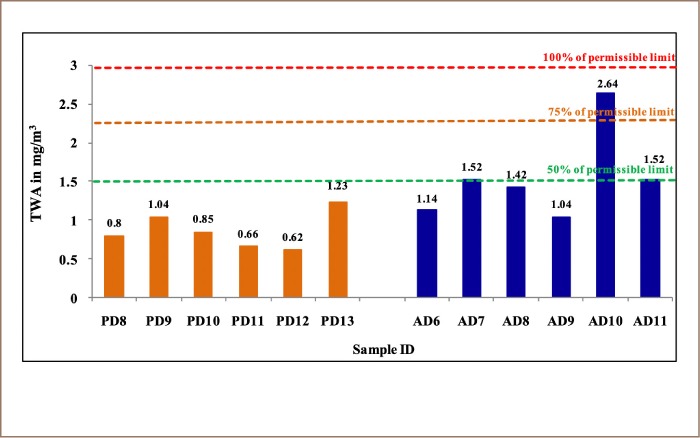
Personal and Area Dust Exposure Monitoring During February 2014

## Discussion

The weather in the month of September 2013 in the mine was temperate and dry. The presence of moisture in the atmosphere after the rainy season suppressed dust levels. The calculated TWA for an 8-hour shift in the workplace for personal and area samples in the mine was below the prescribed limit of 3mg/m^3^ as recommended by DGMS *(Tables 2 and 3).* The September 2013 sampling was conducted just after the rainy season, which could be the reason for low dust concentrations *(Tables 2 and 3).* Based on the results, the operating area for transferring ore at belt conveyor 2 to the pipe conveyer had the highest dust levels compared to all other personal monitoring stations *(Table 2).* Additionally, the crusher hopper area had the highest dust levels compared to all other monitoring stations *(Table 3).*

Dust concentrations were much higher for the majority of personal and area monitoring stations when re-evaluated during the pre-monsoon period in February 2014. In an earlier study, exposure to dust was linked to crushing and drilling operations, which were the activities with the highest dust production, resulting in a significant increase of dust levels in the mine.^13^ As seen from the samples collected in February 2014, the operator of the drilling machine (‘Personal Dust 13’) was found to be more highly exposed to dust compared to others in the mine *(Table 6).* Workers engaged with loading operation tasks (‘Personal Dust 9’) had significant dust exposure, although below permissible limits *(Table 6).* In the examination of area locations, the highest dust exposure (>75% of acceptable limit) was seen at the crusher belt conveyor junction (belt conveyor 1 to belt conveyor 2) *(Table 7).* While fewer workers were needed at the belt area of the crusher conveyor, efforts should be made to reduce the airborne respirable dust concentration at this location. Although the TWA dust concentration at none of the monitoring stations exceeded the permissible limit (3 mg/m^3^) throughout the monitoring period, the probability of exposed individuals suffering health effects after a lifetime of exposure cannot be ruled out. Moreover, the locations where the TWA dust concentrations were found to exceed 50% and 75% of the allowable limit, and work should be halted or limited until major precautions are taken to minimize or inhibit dust generation and inhalation.

It is also probable that the crushing and drilling processes, subsequent transportation and handling of ores generate dust which is rich in silica. Individuals working at these locations are at risk of silica exposure from mine dust. Although the free silica content was below the prescribed limit, there is a possible risk of pneumoconiosis for workers engaged in such activities over prolonged periods.

Guidelines stipulate that mine management should ensure that workers are not exposed to respirable crystalline silica dust concentrations above 0.1 mg/m^3^ (8-hour TWA) i.e. 3.33%.^14^ In the present study, if the free silica results compared with circular No. 01 as above, then the mine situation may be more concerning. However, as per Metalliferous Mines Regulations 124, all the samples analyzed for free silica content by FTIR in September 2013 and February 2014 had free silica present below the prescribed limit of 5%.^15^ Therefore, the threshold limiting value for 8-hour TWA concentrations of airborne respirable dust in the mines based on the present study was 3 mg/m^3^. Prior studies have proposed a trend between silica percentage and respirable dust concentration, i.e. lower dust concentration corresponds with higher variability in silica percentage.^8^ The present study results fit well within this trend.

There is a need to control and maintain effective mining operations where workers are not directly exposed to high dust concentrations. A review of the evidence on free silica dust exposure suggests that chronic lower level exposure to silica poses a risk to human health.^9^ Further research is needed to examine other potential risks from prolonged low-level silica exposure.

The findings of this study may be applied to other similar mines and industries in India and abroad to evaluate dust and free silica exposure risk factors in order to improve work environments.

## Conclusions

The objectives of the present study were to assess respirable dust concentrations in major working areas, personal dust exposure and associated occupational health risks, and free silica content across seasons in an Indian mine. The study was limited to 24 dust samples. A more complete picture may be available with a larger scale study. The 8-hour TWA concentrations of airborne respirable dust in all of the sampled locations for the study conducted in September 2013 and February 2014 were within the limits prescribed by DGMS (3 mg/m^3^). Although all TWA dust concentrations were below 3 mg/m^3^, at some monitoring stations the concentrations exceeded 50% and 75% of the stipulated limit. Dust concentrations at most of the monitoring stations reached a maximum during the pre-monsoon period and were lower in the post-monsoon period. The operations at the crusher belt conveyor junction, blasting, drilling, loading, shoveling and transportation areas were found to have the highest dust levels of all mining operations. The operator near the crusher belt conveyor junction (crusher belt 1 to crusher belt 2) had a greater risk of dust exposure. Drill, dumper, and shovel operators were also found to be more highly exposed during active mining operations. During respirable dust sampling, the crusher plant and mining operation were not active for the full 8-hour shift and the respirable dust TWA average level may not be a representative sample. The free silica content in all of the samples screened during the study period was found to be less than 5% of the permissible limit. However, the exposure of a large number of workers to crystalline silica suggests that further work is needed to develop best practices and strict maintenance to protect workers from the risk of silica exposure.

Prolonged exposure to dust can lead to respiratory disease and serious health problems such as pneumoconiosis, dermatitis, irritation and inflammatory lung injuries, occupational asthma, etc. Safety management along with quantitative assessment of dust exposure can play an important role in reducing dust concentrations at vulnerable sites. Mine officials should implement control measures and monitor airborne respirable dust in the mine. These continued efforts can lead to more eco-friendly mining and a better work environment for all workers in the area.
